# Characterization and Evaluation of a Novel Conserved Membrane Antigen P35 of *Mycoplasma synoviae*

**DOI:** 10.3389/fvets.2022.836110

**Published:** 2022-02-23

**Authors:** Qianjin Sun, Xiaona Wei, Wei Chen, Qian Zhong, Zhuanqiang Yan, Qingfeng Zhou, Yongchang Cao, Feng Chen

**Affiliations:** ^1^College of Animal Science, South China Agricultural University, Guangzhou, China; ^2^State Key Laboratory of Biocontrol, School of Life Sciences, Sun Yat-sen University, Guangzhou, China; ^3^Guangdong Enterprise Key Laboratory for Animal Health and Environmental Control, Wen's Foodstuff Group Co., Ltd., Yunfu, China; ^4^Wen's Group Academy, Wen's Foodstuffs Group Co., Ltd., Xinxing, China

**Keywords:** *Mycoplasma synoviae*, membrane protein, novel antigen, immunogenicity, antigenicity

## Abstract

*Mycoplasma synoviae* (MS) is a major avian pathogen that causes respiratory damage, infectious synovitis, and arthritis in chickens and causes serious economic losses to the global poultry industry. Despite its significance, knowledge on pathogenicity and pathogenic mechanism of MS is lacking, especially regarding its antigens. Bioinformatic analysis showed that the known MS proteins are only the tip of the iceberg among many MS membrane proteins. In this study, we identified and expressed a novel MS membrane protein P35. Sequence similarity showed that P35 was conservative and commonly existed among MS strains. Membrane protein extraction and immunofluorescence assay confirmed that P35 was distributed on the surface of MS. The production of specific antibodies after immunization with recombinant protein rP35 suggested its immunogenicity. The antigenicity of P35 was evaluated from two aspects by using polyantiserum against MS and rP35. Furthermore, in assays to identify the immune peptides of P35, all successfully expressed truncated segments could react with positive polyantiserum of MS, suggesting that P35 had more than one immune peptide. In conclusion, our study successfully identified P35 as a conservative antigen of MS, which may act as a potential candidate for the future development of a vaccine against MS.

## Introduction

*Mycoplasma synoviae* (MS) is an important pathogen in the poultry industry. MS infection can cause subclinical to acute and chronic respiratory damage, infectious synovitis, and arthritis in chickens (1–3). Although, MS infections rarely cause direct death of chickens, they lead to obvious lameness, growth retardation, lower availability of ketone bodies, lower egg production rate, and higher eggshell apex abnormalities, leading to serious economic losses in the poultry industry (4–6). Besides, MS co-infections with other infectious agents such as Newcastle disease virus, *Escherichia coli*, and *Mycoplasma gallisepticum* increase economic losses (7–10).

With the emergence of drug-resistant strains (11–13), vaccination is the most effective way to control MS, combined with hygiene and management procedures (14, 15). However, current vaccines are not able to prevent transmission of MS (16, 17) and are expensive, as *in vitro* growth requires a rich medium and is time-consuming (18, 19). The development of more effective and cheaper vaccines requires to identify and characterize antigens of MS. Also, investigation on pathogenicity mechanism and serological detection methods are hampered by the lack of known and well-characterized MS antigens. However, only a few antigens have been identified so far, such as lipoprotein MSPB, elongation factor EF-Tu, enolase, NADH oxidase, hemagglutinin MSPA, ATP synthase beta chain, trigger factor, pyruvate kinase, chaperone DnaK, and pyruvate dehydrogenase complex E1 alpha and beta subunits (20, 21). But the immunogenicity and antigenicity of these proteins have not been thoroughly studied. Our analysis of the MS genome showed that there were still a large number of hypothetical antigens that had not been identified, and more research is needed on MS immunogenic proteins.

Mycoplasma lack cell walls, and since the cytoplasmic membrane components are directly in contact with host cells, they are generally thought to play an important role in survival, replication, and virulence compared to those in other bacterial species with cell walls (22); however, many mycoplasma proteins remain hypothetical (23). Many mycoplasma gene products have little or no detectable sequence similarity to those characterized in other bacteria (24). Improved understanding of the molecular pathogenesis of mycoplasmas will thus depend on the characterization of the function of these proteins that appear to be specific to the mycoplasmas (25, 26). Experimental validation of the functions of surface proteins of mycoplasma has been identified as one of the critical gaps in understanding this pathogen (27).

## Materials and Methods

### *In silico* Analysis of *Mycoplasma synoviae* Genome

Bioinformatic analysis of MS genome (WVU1853, NZ_CP011096.1) was carried out with several online resources. Without the cell wall, membrane proteins of MS were the focus of analysis. Psortb v3.0 was used to detect cellular location (cytoplasmic, cytoplasmic membrane, outer membrane, or extracellular) (28). BOMP was used to detect integral β-barrel outer membrane proteins (29). Lipoprotein and signal peptides were analyzed by LipoP 1.0 (http://www.cbs.dtu.dk/services/LipoP/). HMMER 3.2.1 software and Pfam v32.0 database were used to identify the functional domain of proteins (30). Lipoproteins with strong β-barrel signaling on the cell membrane were identified as potential immunogenic proteins. Then these proteins were subjected to functional domain identification and functional classification. A total of 149 membrane-associated proteins were preliminarily identified. Based on proteins scores in each prediction, we selected 27 proteins for further study. Of these 27 proteins, eight were hypothetical proteins, and P35 was one of them.

For the bioinformatic analysis of hypothetical protein P35, amino acid sequence similarity was compared by Basic Local Alignment Search Tool (BLAST) analysis at the National Center for Biotechnology Information (NCBI; http://www.ncbi.nlm.nih.gov). Physical data such as theoretical pI, molecular weight, and extinction coefficients were collected using the ProtParam tool at ExPASy (https://www.expasy.org/) (31). Theoretical transmembrane domain and signal peptide scores were identified using the TMHMM (http://www.cbs.dtu.dk/services/TMHMM/) and SignalP (http://www.cbs.dtu.dk/services/SignalP/) web services, respectively. B-cell epitopes of P35 were predicted using Immune Epitope Database (IEDB) (http://tools.iedb.org/main/). As TGA codons act as terminators in *E. coli* and as tryptophan codons in mycoplasmas, proteins with more than five TGA-coded tryptophans were excluded.

### Gene Homology Analysis

Due to the lack of gene sequences of *p35* in the NCBI database, we designed and synthesized specific primers to amplify and sequence *p35* genes from MS isolated strains. The homology of *p35* genes was calculated using MegAline with the ClustalW method.

### Cloning, Expression, and Purification of Recombinant Protein

To obtain the P35, the pSYNO-1 expression vector with chloramphenicol resistance site containing the target CDS was synthesized by Convenience Biology (Changzhou, China). The TGA codon contained in CDS was changed to TGG in the DNA sequence sent for synthesis. *E. coli* pGro7/BL21(DE3) cell (BJBALB, Beijing, China) was used for protein expression. For the solubility test, 50 ml of LB containing 100 mg/ml of kanamycin and 25 μg/ml of chloramphenicol was inoculated with 500 μl of pGro7/BL21(DE3) and incubated at 37°C with shaking to the optical density 600 (OD600) >0.600. Then, 50 μg/ml of IPTG was added to the broth culture. Induction expression was carried out at 25°C overnight. The culture was centrifuged, and the pellet was homogenized with 0.1 M of Tris-HCl. Cells were lysed by sonication for 2 min, 4 s on and 4 s off pulse, 30% of amplitude (JY92-IIDN, SCIENTZ, Ningbo, China). Final samples were centrifuged, and the pellets homogenized in a suitable buffer. The presence of the MBP-tagged proteins in all fractions was determined using sodium dodecyl sulfate–polyacrylamide gel electrophoresis (SDS-PAGE) followed by Western blotting using anti-MBP (maltose-binding protein) antibody.

For protein purification, the supernatant of lysed samples was collected and purified in a nickel chelating resin (High Affinity Ni-Charged Resin FF Prepacked Column, GenScript, Nanjing, China) under suitable conditions. Chromatographic fractions were collected using an imidazole gradient and visualized in SDS-PAGE. Protein concentrations were determined using NanoPhotometer® NP80 (IMPLEN, Westlake Village, CA, USA).

### Production of Polyclonal Antibodies of Recombinant Protein

The purified protein rP35 or MBP was diluted in oil adjuvant (Beyotime, Shanghai, China) at a ratio of 2:3 according to the manufacturer's instructions. Twelve 10-day-old specific pathogen-free (SPF) chickens were used to raise serum against rP35 and MBP. Two doses of purified protein were administered by subcutaneous injection at a 14-day interval. Blood samples were collected from the wing vein at 0, 7, 14, 21, and 28 days after the first inoculation (DAI), and isolated serums were stored at −80°C before being processed. All animal experiments were supervised by the Institutional Animal Care and Use Committee of Sun Yat-sen University (SYSU-IACUC-2021-B0507) and used in accordance with the regulation and guidelines of this committee.

### Antigenicity Assays of Chimeric Protein

To identify whether P35 has antigenicity, ELISA and Western blotting assay were performed. For ELISA, microtiter plates were coated with 40 μg/well of purified protein or 10^8^ CCU/well of MS isolated strain named SD2. Serum samples of purified protein and MS SD2 strain originating from SPF chickens were used as primary antibodies with dilution at a ratio of 1:1,000. The negative serum samples were obtained from animals without vaccine or infection. Horseradish peroxidase (HRP)-conjugated goat anti-chicken IgG (KPL, USA) was used as a secondary antibody with 1:5,000 dilution. For Western blotting assay, the same antibodies were used as those in ELISA.

### Immunofluorescence Assays

The localization of P35 on the MS surface was determined using immunofluorescence assays. MS SD2 cells were collected at the mid-logarithmic phase by centrifuging at 5,000 × g for 5 min and washed three times with phosphate-buffered saline (PBS). The cell pellets were resuspended in PBS containing 5% (w/v) skim milk and incubated for 1 h at 37°C. After centrifugation and washing, cells were resuspended in polyantiserum of rP35 (1:500) or MBP (1:500) and incubated for 1 h at 37°C. Cells were washed three times and incubated with fluorescein isothiocyanate (FITC)-conjugated goat anti-chicken IgG (1:1,000, KPL) at 4°C for 1 h. The cells were washed three times, spread onto glass slides, and observed by fluorescence microscope (TCS SP8, Leica, Wetzlar, Germany).

### Extraction of Membrane Protein

MS membrane proteins were extracted using Bacterial Membrane Protein Extraction Kit (KALANg, Shanghai, China) according to the manufacturer's instruction. Briefly, MS SD2 cells were collected at the mid-logarithmic phase by centrifuging at 5,000 × g for 5 min and washed twice with PBS. Then 500 μl of extracting solution A was added to cells and vortexed fully at 4°C for 1 h until cells were completely lysed. The lysate was centrifuged at 4°C 12,000 × g for 5 min. The supernatant was collected and incubated at 37°C for 10 min and then centrifuged at 1,000 × g for 3 min. The top solution was a mixture of cytoplasmic proteins, and the bottom solution was a mixture of membrane proteins. The membrane proteins were dissolved with 100 μl of cold solvation reagent. The samples of cytoplasmic proteins and membrane proteins were stored at −80°C for later use.

### Immune Epitope Region of P35

To identify which region of P35 has antigenicity, we divided the protein into five segments for truncated expression, named SP1, SP2, SP3, SP4, and SP5. The expression vector and purification process were the same as those of rP35. SDS-PAGE and Western blotting assays verified the expression of truncated proteins. ELISA and Western blotting assay with the polyantiserum of MS as the primary antibody were carried out to confirm the antigenicity.

### Statistical Analysis

Statistical analysis was performed using GraphPad Prism 8.0. The data were initially analyzed by the Kolmogorov–Smirnov test to verify the distribution of the data. To evaluate the reactivity between the recombinant proteins with the chicken antiserum in ELISA, two way-ANOVA was performed when comparing more than two groups. Data were expressed as mean ± SD. Statistical differences were considered significant when *p* < 0.05 using a 95% CI.

## Results

### Bioinformatic Selection and Evaluation of Antigenic Proteins

The genome of the MS WVU1853 strain, obtained from the NCBI database, contains 675 protein coding sequences (CDSs). Among these CDSs, Psortb predicted 149 CDSs to be located on the cytoplasmic membrane, 14 CDSs were extracellular, and 227 CDSs were unknown. After analysis with BOMP, LipoP 1.0, and Pfam, we identified that P35 was located on the cell membrane, was not a lipoprotein, and had signal peptide and was therefore selected for further analysis in this study.

**Figure 1:**
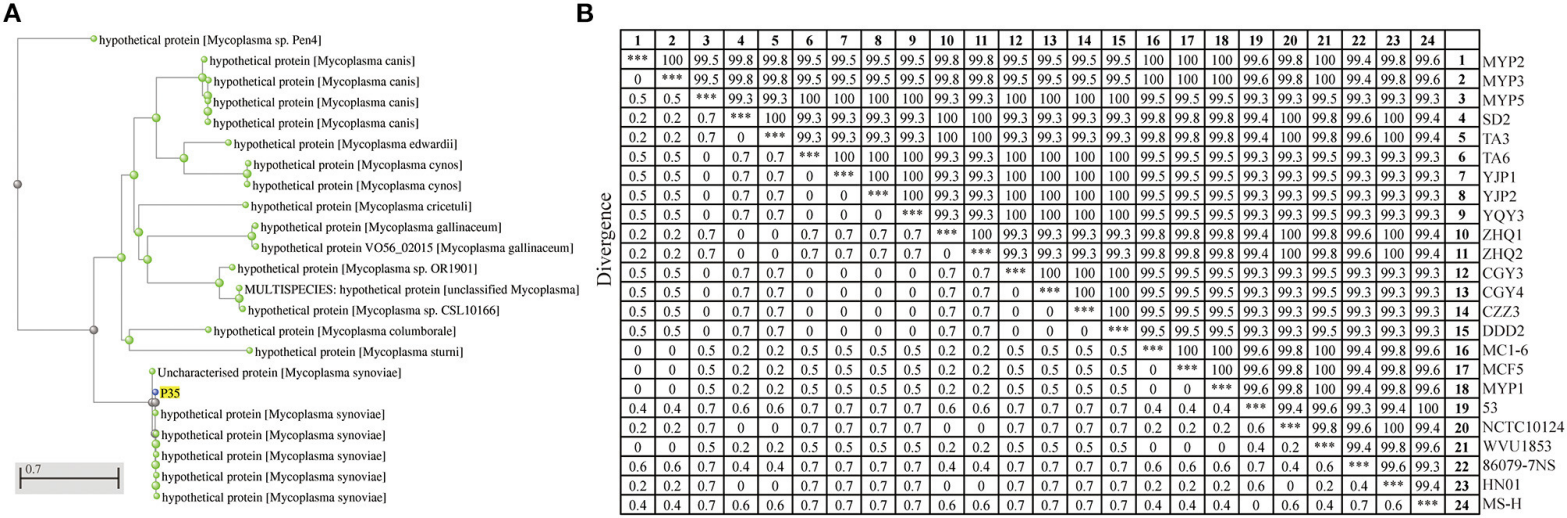
Homology analysis of P35. **(A)** Molecular evolutionary tree analysis of P35. This tree was drawn on National Center for Biotechnology Information (NCBI) after being blasted with amino acid sequences of P35. The green points mean the blasted results of P35, and the blue point is P35. **(B)** Sequence distance of p35 gene among different *Mycoplasma synoviae* (MS) strains. A total of 24 sequences were used to calculate the homology by MegAline, among which 18 sequences were amplified and sequenced from MS isolates, and 6 sequences (53, NCTC10124, WVU1853, 86079-7NS, HN01, and MS-H) were obtained from NCBI database. ***The consistency of the same strain, which means 100% similarity.

Homology detections with BLAST tools showed that P35 had a low similarity with other type mycoplasma proteins; the highest identity among the results was 31.03% with hypothetical protein of *Mycoplasma columborale* (Figure 1A). After ProtParam analysis, the theoretical molecular weight of P35 was 45.788 kDa, and the theoretical pI was 5.71. The TMHMM and SignalP algorithms identified a transmembrane domain from residues 10 to 32 (probability > 0.98) and a signal peptide (likelihood was 0.8983).

### Generality and Conservatism Analysis of p35 Gene

To prove that P35 is a universal protein of MS, the specific amplification primers of *p35* genes were designed and synthesized, and 18 *p35* genes from different MS isolates were amplified and sequenced. A total of 6 *p35* genes (from strains of 53, NCTC10124, WVU1853, 86079-7NS, HN01, and MS-H) were downloaded from the NCBI database. A total of 24 sequences were used for the homology analysis of *p35* gene. As shown in Figure 1B, the identity of *p35* genes was among 99.3% to 100%. These results showed that *P35* is a common and conservative protein on MS.

### Recombinant Protein Expression and Purification

**Figure 2:**
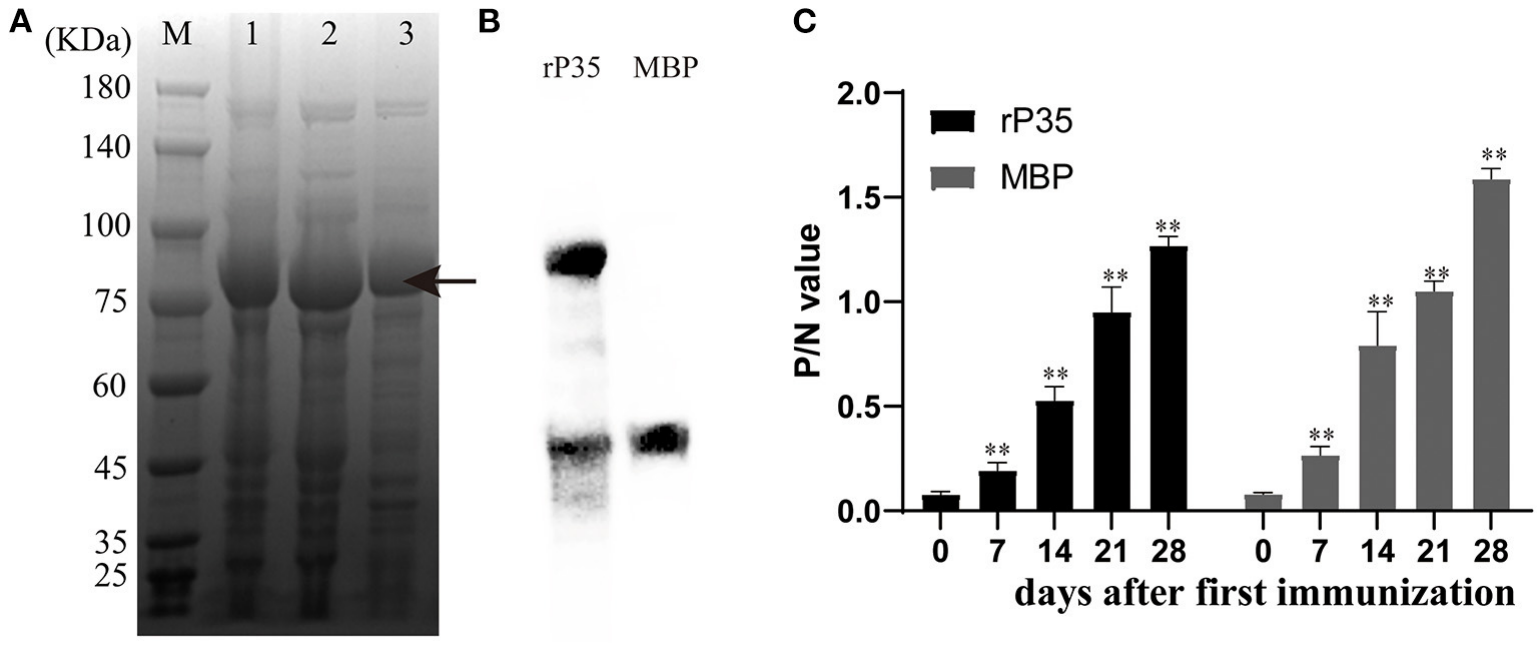
Expression and preparation of polyantiserum of rP35. **(A)** Coomassie Blue staining of the *Mycoplasma synoviae* (MS) protein expressed in *Escherichia coli* BL21 (DE3) and run on 10% polyacrylamide gels. Lane M: standard protein marker. Lane 1: total cell extract of *E. coli* BL21 (DE3). Lane 2: soluble fraction of the total bacterial extract after induction. Lane 3: protein purified by nickel column. The two bands in lane 3 may be the fractured recombinant proteins during purification. **(B)** Western blotting assay to verify the successful expression of recombinant protein. The right lane is MBP protein and the left lane is recombinant protein rP35. The primary antibody used was monoclonal antibody of anti-MBP tag. **(C)** Production of polyantiserum of rP35. rP35- and MBP-specific IgG responses induced in specific pathogen-free (SPF) chickens immunized with protein (400 μg) performed using blood samples collected every 7 days for 28 days. **means there were significant differences; Arrow means the target protein we obtained.

The pSYNO-1 expression vector we used in this study contains the MBP tag, which will increase the molecular weight of the target protein by about 40 kDa. As shown in Figure 2A, the recombinant protein (rP35) was soluble, and the molecular weight was ~87 kDa. The purified recombinant protein presented a single band. Western blotting assay confirmed the successful expression of rP35 (Figure 2B), while the two bands in the right lane may mean the recombinant protein breaks in two, which could not separate for the similar molecular weight of P35 and MBP tag.

### Specific Polyclonal Antibodies Against rP35

SPF chickens aged 10 days were immunized with rP35 or MBP for the first time and were immunized again after 14 days. Serum was collected on days 0, 7, 14, 21, and 28 after the first vaccination and detected simultaneously. The kinetics of antibody production in chickens demonstrated a significant increase after immunization with the antigens rP35 and MBP compared to the pre-immune serum (day 0) (Figure 2C). These results suggested that recombinant protein rP35 had immunogenicity and could stimulate the production of specific antibodies in chickens.

### Antigenicity of Recombinant Proteins

**Figure 3:**
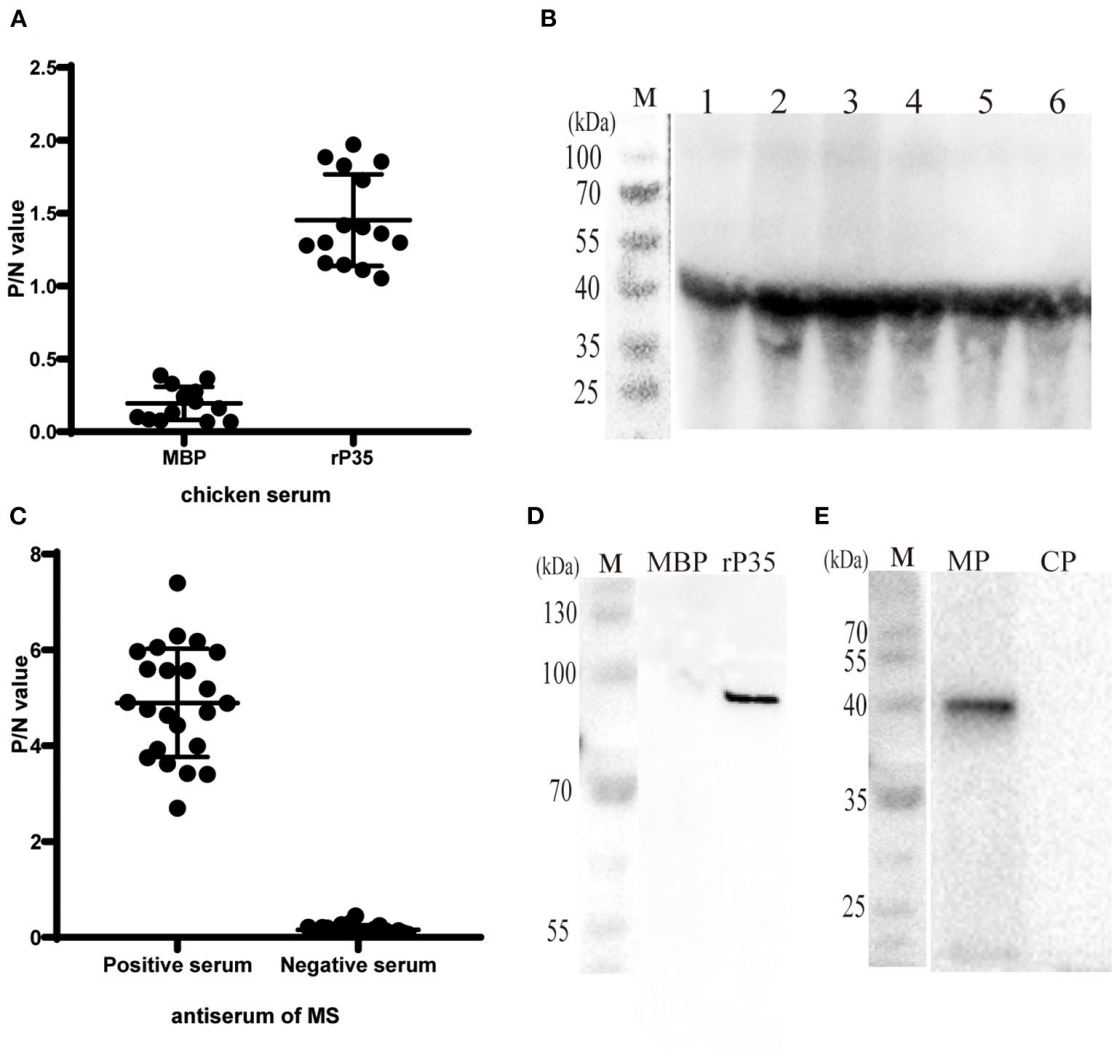
Antigenicity analysis and localization of P35. **(A)** ELISA plates were coated with different *Mycoplasma synoviae* (MS) strains (10^8^ CCU/well) and incubated with chicken serum against MBP and rP35 as the primary antibodies. **(B)** Western blotting assay of different MS strains incubated with chicken serum against rP35. **(C)** ELISAs were coated with rP35 and incubated with positive and negative specific pathogen-free (SPF) chicken serum against MS as the primary antibodies. **(D)** Western blotting assay of MBP and rP35 incubated with positive SPF chicken serum against MS. **(E)** Localization of P35. Western blotting assay was carried out after extracting the membrane protein of MS. MP means the membrane protein and CP means the cytoplasmic protein. The primary antibody used was SPF chicken serum against rP35.

To clarify whether P35 has antigenicity, forward and reverse assays were carried out. On the one hand, we used SPF chicken polyantiserum of rP35 and MBP as primary antibodies to react with different MS strains in both ELISA and Western blotting assay. The results showed that significantly higher P/N values occurred between antiserum of rP35 and MS strains, and the values of P/N between antiserum of MBP and MS strains were of nearly basal level (Figure 3A). Correspondingly, a specific band can be observed obviously in Western blotting results (Figure 3B). On the other hand, we tested the reactivity of rP35 with antiserums of MS. In ELISA, positive and negative chicken serum samples of anti-MS were used to react with recombinant protein. The P/N values of positive serum samples were significantly higher than those of negative serum samples (Figure 3C). The results of Western blotting showed that the recombinant protein was recognized by positive anti-MS chicken serum, and there was no band in the lane of MBP (Figure 3D). These results proved that P35 protein is antigenic.

### Cellular Localization of the P35

**Figure 4:**
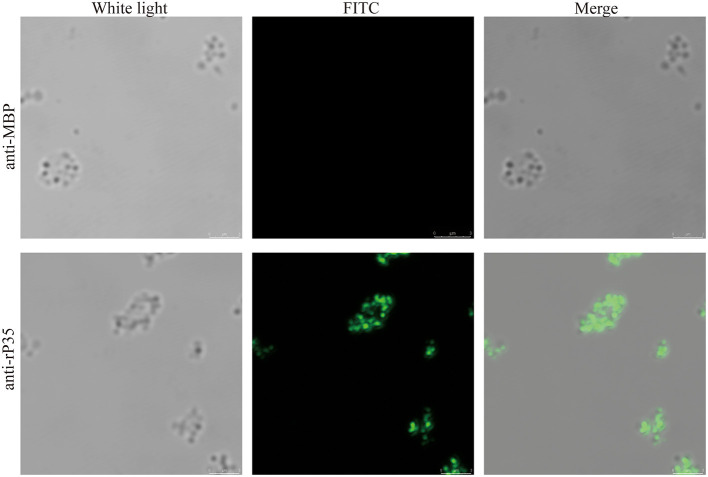
Immunofluorescence assays of P35. After being washed twice, *Mycoplasma synoviae* (MS) cells were incubated with specific pathogen-free (SPF) chicken serum against rP35 and MBP without being permeated. Secondary antibody was fluorescein isothiocyanate (FITC)-conjugated goat anti-chicken IgG. Scale bars indicate 3 μm.

Although bioinformatic analysis showed that P35 was an MS membrane protein, it was necessary to confirm this conclusion by molecular biology method. We extracted the membrane protein of MS with a commercial kit. The obtained membrane protein mixture and cytoplasmic protein mixture were subjected to Western blotting with rP35 antiserum as the primary antibody. The results showed that a specific band was observed in the membrane protein lane (Figure 3E). Besides, we visually observed whether P35 was located on the cell membrane by fluorescence microscope in a state where the cells were not being permeated. As expected, a strong green fluorescent signal was observed under the microscope (Figure 4). In short, these studies suggested that p35 was located on the cell membrane.

### Immune Epitope Region of P35

**Figure 5:**
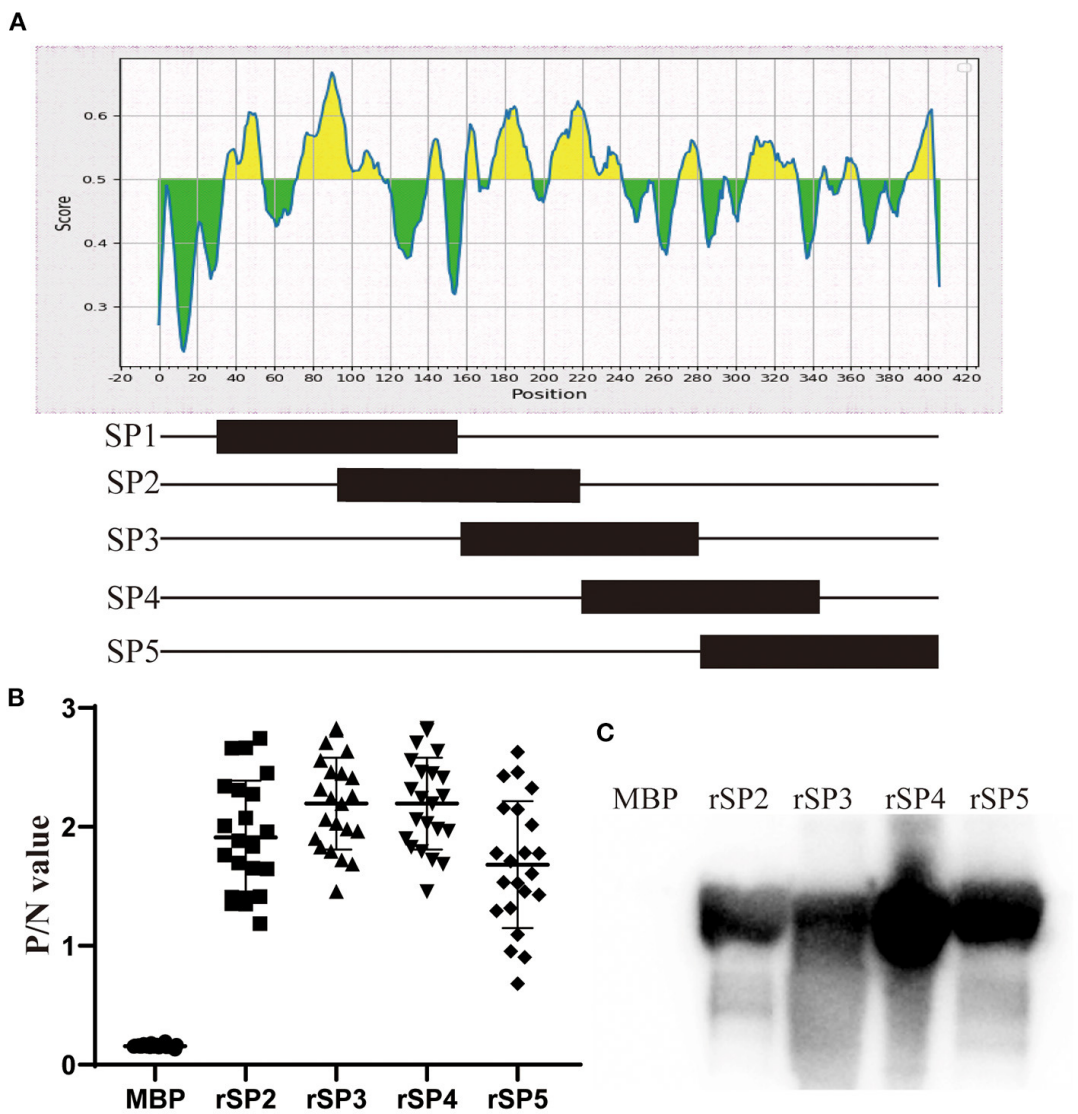
Immune epitope analysis of P35. **(A)** Schematic diagram of truncated expression of P35. Excluding the signal peptide region, P35 protein was divided into five segments, and half the sequence repeats are between adjacent segments. **(B)** ELISAs were developed by coating plates with rSP2, rSP3, rSP4, and rSP5 segments and incubated with chicken serum against *Mycoplasma synoviae* (MS) as primary antibody, and MBP was coated as the negative control. **(C)** Western blotting assay of rSP2, rSP3, rSP4, and rSP5 segments reacted with chicken serum against MS. MBP was used as the negative control.

In order to identify which regions of P35 have antigenicity, we truncated protein into five segments and expressed as SP1–SP5 (Figure 5A). Purification of the SP1 segment failed (data not shown). Thus, SP2–SP5 segments were used to test the antigenicity. In the ELISA, truncated segments were coated at the same concentration.

The results showed that the P/N values of four truncated segments, reacting with chicken antiserum of MS, were higher than 0.681, while values of MBP reacted with antiserum were lower than 0.172 (Figure 5B). Western blotting assay also approved the specific reaction between four truncated segments and antiserum of MS, and no band was observed in the lane of MBP (Figure 5C). These results suggested that the P35 protein has several immune epitopes, which are dispersed in various regions of the protein.

## Discussion

MS is a major avian pathogen and is one of the main pathogens causing respiratory tract infection and joint swelling in chickens. MS is distributed worldwide and causes serious economic losses to the global poultry industry every year. Research on MS can lay the foundation for the prevention, diagnosis, and treatment of MS-related diseases (20). Previous studies have screened several immunogenic proteins from MS isolates (21). Bioinformatic analysis showed that there are still a large number of MS proteins that have not been identified, some of which are immunogenic proteins. In the present study, we identified a novel MS protein (P35) that has immunogenicity and antigenicity. To our knowledge, this was the first study to propose P35 in MS even in mycoplasma, which limits the speed of in-depth research on the structure, function, and so on due to the lack of relevant studies as a reference.

Firstly, we aligned the P35 protein with BLASTp and hoped to find some additional information about it. However, no known protein was aligned (Figure 1A). Then, we analyzed the sequence similarity of *p35* genes of different MS strains. It was found that *p35* genes were conservative, and the lowest sequence identify was 99.3% (Figure 1B). Therefore, we determined that P35 had value for further study.

For a novel conserved protein, one of our primary concerns is whether P35 is an antigen of MS. To verify it, we expressed rP35 and raised its polyantiserum. After immunization with rP35, chickens produced specific antibodies (Figure 2C), which suggested that rP35 has immunogenicity. Then we designed assays to identify the antigenicity of P35 (Figure 3). Firstly, we let the rP35 and MBP react with the chicken positive anti-MS serum samples in, respectively, ELISA and Western blotting assay. Significant reactivity was detected between rP35 and antiserums rather than MBP. Then, we used antiserum of rP35 and MBP to react with different MS isolated strains. As expected, a positive reaction occurred between MS strains and antiserum of rP35. At this point, we proved that P35 had immunogenicity and antigenicity and was an antigen of MS. However, whether immunization with P35 can protect chickens against MS infection needs further study.

The molecular weight of expressed recombinant protein indicated in Western blotting analysis is 87 kDa, which is consistent with the estimated molecular weight by bioinformatic analysis; however, there have been other 40-kDa protein bands. It is common especially for large proteins to have multiple bands due to protein truncation (32, 33). Truncated proteins are due to protein degradation or premature termination in ribosomes. Several studies on the expression of recombinant MBP fusion proteins faced this issue and considered it as a normal situation (34, 35).

In the present study, we also designed assays to express truncated segments to identify the immunogenic peptides of P35. However, four of the five truncated segments were successfully expressed, and all of the four segments could react with positive anti-MS serum samples. This result indicated that more than one immune peptide existed in P35.

In conclusion, in the present study, we identified a novel conserved membrane protein of MS. Furthermore, immunological studies confirmed that P35 protein had immunogenicity and antigenicity and was an MS antigen, which means that P35 may be a potential candidate against MS. However, other characteristics of P35 such as the molecular function, spatial structures, and immune protection need to be further studied.

## Data Availability Statement

The original contributions presented in the study are included in the article/supplementary material, further inquiries can be directed to the corresponding author/s.

## Ethics Statement

The animal study was reviewed and approved by the Institutional Animal Care and Use Committee of Sun Yat-sen University (SYSU-IACUC-2021-B0507).

## Author Contributions

XW and FC analyzed the genome of MS and designed this study. QS and WC prepared the recombinant protein. QZhong and ZY did the immunogenicity analysis of the protein. XW wrote the manuscript. QZhou, YC, and FC revised and corrected it. All authors contributed to the article and approved the submitted version.

## Funding

This work was supported by the Guangdong Basic and Applied Basic Research Foundation (2019A1515012006), Key Research and Development Program of Guangdong Province (2020B020222001), and China Postdoctoral Science Foundation (2021M692455).

## Conflict of Interest

XW and YC were employed by Wen's Foodstuffs Group Co., Ltd. The remaining authors declare that the research was conducted in the absence of any commercial or financial relationships that could be construed as a potential conflict of interest.

## Publisher's Note

All claims expressed in this article are solely those of the authors and do not necessarily represent those of their affiliated organizations, or those of the publisher, the editors and the reviewers. Any product that may be evaluated in this article, or claim that may be made by its manufacturer, is not guaranteed or endorsed by the publisher.
